# Supplementary material for: Extensive Variation in Thermal Responses and Toxin Content Among 40 Strains of the Cold-Water Diatom Pseudo-nitzschia seriata—In a Global Warming Context
Source: Toxins (Basel). 2025 May 9;17(5):235. doi: 10.3390/toxins17050235 (PMC12115825; doi:10.3390/toxins17050235)
Supplement: Supplementary file 1 [file toxins-17-00235-s001.zip › toxins-3582186-supplementary.pdf]

Supplementary Materials: Extensive Variation in Thermal Responses and Toxin Content Among 40 Strains of the Cold-Water Diatom *Pseudo-nitzschia seriata*—In a Global Warming Context

Caroline Weber, Anna Junker Olesen, Robert G. Hatfield, Bernd Krock and Nina Lundholm

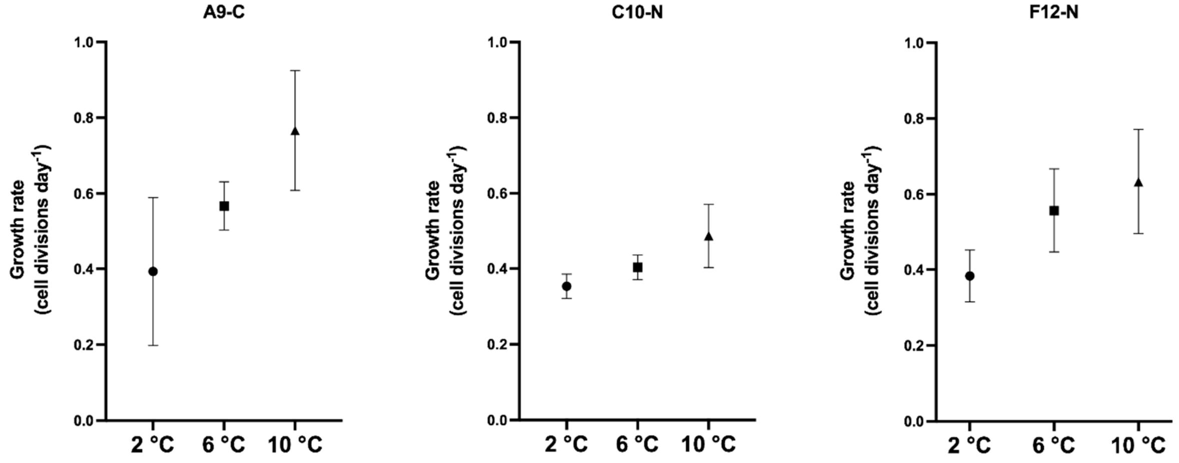

Figure S1. Mean (± SD) GR (day^{-1}) for the three triplicate strains (A9-C, C10-N and F12-N) of *P. seriata* at temperatures 2 °C, 6 °C and 10 °C.

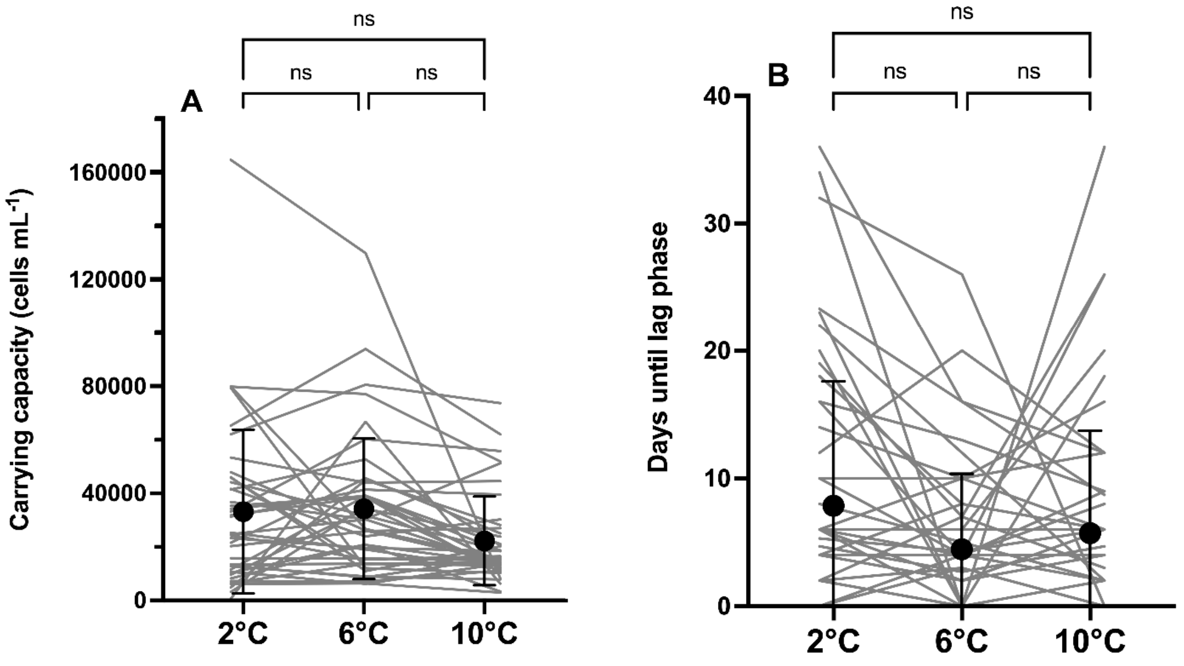

Figure S2. Carrying capacity (cells mL^{-1}) (A), and lag phase (B) at temperatures 2 °C, 6 °C and 10 °C. Significance between temperatures is shown as ns = no significance.

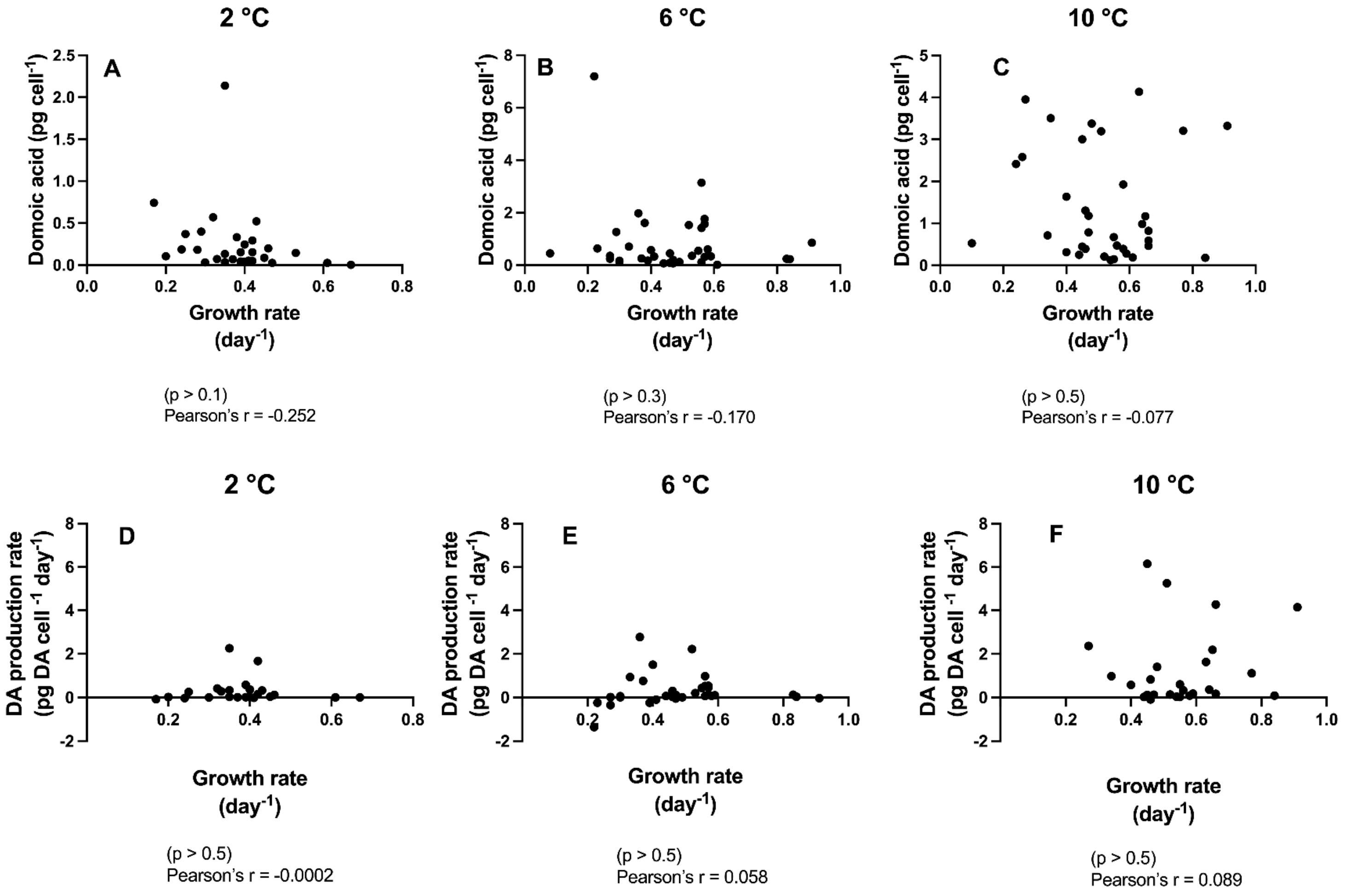

Figure S3. Correlations between growth rate and domoic acid content (pg cell^{-1}) (A-C) and between growth rate and cellular domoic acid production rate (pg DA cell^{-1} day^{-1}) in exponential phase (D-F). P-value and Pearson's r-value is listed beneath each graph. Note different y-axes.

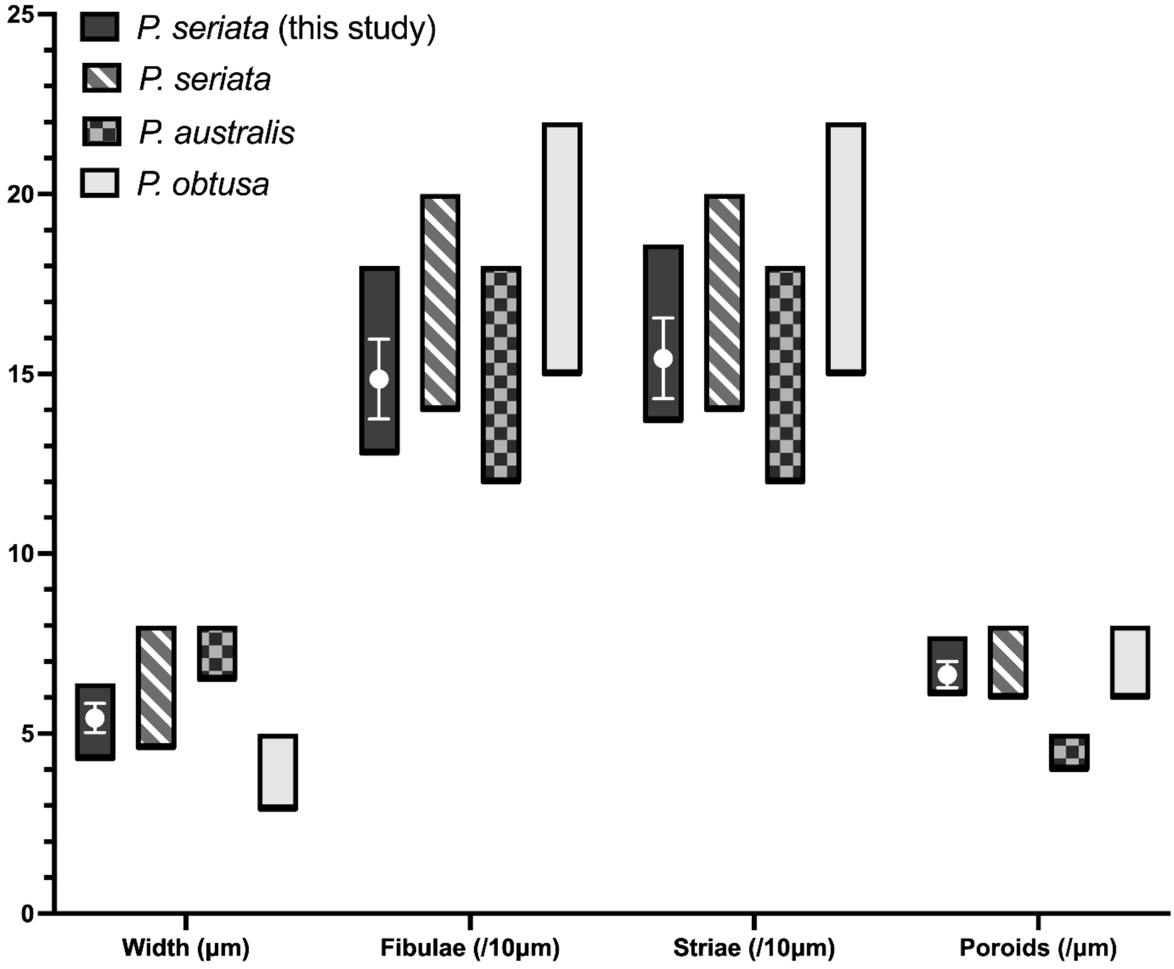

Figure S4. Phenotypic morphological variation seen as boxplots comparing the range (min./max.) of five morphological characters of *P. seriata* (28 strains) with the accepted ranges of *P. seriata, P. australis* and *P. obtusa* (based on Hasle & Lundholm [55] and references herein). White dots with error bars indicate the mean (±SD) of *P. seriata* characteristics in the strains from the current study.

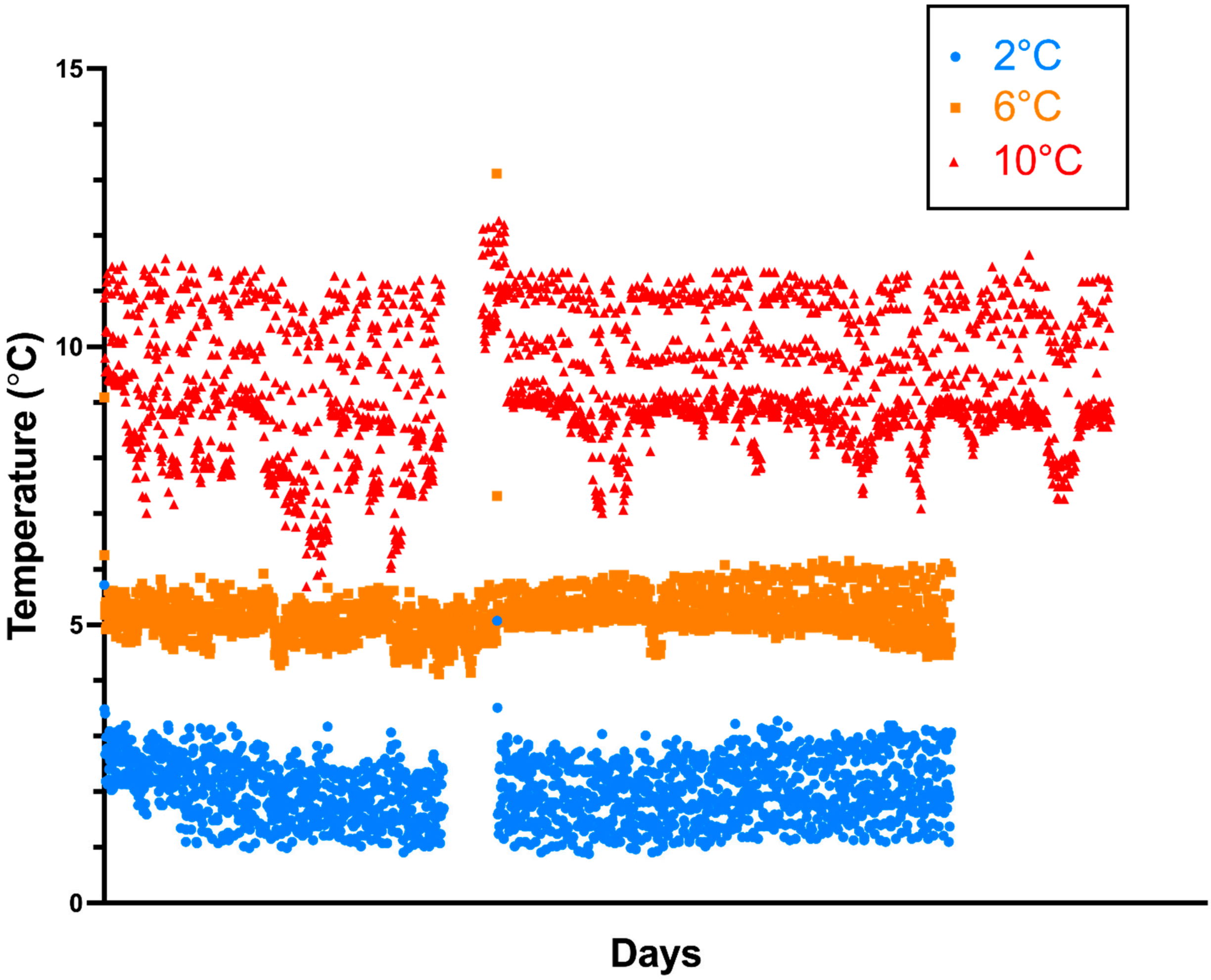

Figure S5. Temperature (°C) logged in the three chambers. Blue dots correspond to temperatures logged in the 2 °C-chamber, yellow dots correspond to temperatures logged in the 6 °C-chamber and red dots correspond to temperatures logged in the 10 °C-chamber.

Table S1. Domoic acid levels (pg DA cell^{-1}) in exponential and stationary phases at three temperatures in 40 strains of *Pseudo-nitzschia seriata.*

	2 °C		6 °C		10 °C	
	Exponential	Stationary	Exponential	Stationary	Exponential	Stationary
Mean ± SD	0.20 ± 0.29	0.28 ± 0.38	0.81 ± 1.13	0.50 ± 0.57	1.47 ± 1.53	1.61 ± 2.03
Minimum	0.004	0.002	0.01	0.01	0.09	0.04
Maximum	2.14	1.96	7.20	2.49	8.37	9.84

Table S2. Overview of genetic differences in 18S, ITS and 28S rDNA of 52 strains of *Pseudo-nitzschia seriata* isolated from the same water sample, note: nucleotide in brackets for variations in position 2239 identify the dominant variant in strain. Additional sequences (ITS1, 5.8S and ITS4) obtained from Genbank of nine strains is included. Grey represents positions in 18S and 28S which was not included in the Genbank-sequences.

Location	Strain	Accession no.	1422	2006	2239	2946
Denmark	A9-C		C		T	G
Denmark	A1-N		C		T	G
Denmark	A2-N		C		Y (T)	G
Denmark	A3-N		C		T	G
Denmark	A6-N		C		Y (C)	G
Denmark	A8-N		C		Y (C)	G
Denmark	A10-N		C	GCCC inserted	T	G
Denmark	B4-N		C		T	A
Denmark	B6-N		C		Y (T)	G
Denmark	B7-N		C		Y (T)	G
Denmark	B8-N		C		T	G
Denmark	B9-N		C		Y (T)	G
Denmark	B11-N		C		T	G
Denmark	B12-N		C		T	G
Denmark	C3-N		C		T	G
Denmark	C5-N		C		Y (T)	G
Denmark	C6-N		C		T	G
Denmark	C7-N		C		T	G
Denmark	C8-N		C		Y (T)	G
Denmark	C10-N		C		Y (T)	G
Denmark	D1-N		C		Y (T)	G
Denmark	D3-N		C		Y (T)	G
Denmark	D8-N		C		T	G
Denmark	D10-N		C		T	G
Denmark	D11-N		C		T	G
Denmark	E3-N		C		T	G
Denmark	E4-N		C		T	G
Denmark	E6-N		C		T	G

Denmark	F2-N		C	Y (T)	G
Denmark	F3-N		C	T	G
Denmark	F6-N		C	T	G
Denmark	F7-N		C	T	G
Denmark	F8-N		C	T	G
Denmark	F9-N		C	T	G
Denmark	F10-N		C	T	G
Denmark	G2-N		C	T	G
Denmark	G3-N		C	T	G
Denmark	H1-N		C	Y (T)	G
Denmark	H9-N		C	T	G
**Not in experiment**					
Denmark	A11-N		C	T	G
Denmark	B2-N		C	T	G
Denmark	C1-N		C	T	G
Denmark	C4-N		C	T	G
Denmark	D2-N		C	T	G
Denmark	D4-N		C	T	G
Denmark	E5-N		C	T	G
Denmark	E9-N		C	T	G
Denmark	F1-N		A	T	G
Denmark	G5-N		C	Y (T)	G
Denmark	G12-N		C	Y (T)	G
Denmark	H4-N		C	T	G
**Genbank**					
Greenland	A3	JF974030		T	
Greenland	A2	JF974031		T	
Greenland	C1	JF974032		T	
Greenland	C2	JF974033		T	
Denmark	Lynaes6	DQ062663		T	
Denmark	Lynaes8	DQ062666		T	
Scotland	PLYSt16B	AY452523		T	
Scotland	PLYSt52B	AY452524		T	
Denmark	Nissum3	AY257841		Y (C)	

Table S3. Domoic acid production rate (pg DA cell^{-1} d^{-1}) in exponential- and stationary growth phase for 40 strains of *P. seriata* at temperatures 2°C, 6°C and 10°C. For strains A9-C, C10-N, and F12-N mean and standard deviation is shown. ND: no data, NG: no growth/no clear growth phases, NS: no sample, *: content lower than 0.00 but with detectable DA content.

	2 °C		6 °C		10 °C	
Strain	Exponential	Stationary	Exponential	Stationary	Exponential	Stationary
A9-C	NG	NG	0.45 ± 0.06	0.04 ± 0.04	1.11 ± 0.27	-0.42 ± 0.14
A1-N	0.04	-0.00*	-0.03	0.39	1.92	2.06
A2-N	0.02	0.02	0.06	0.03	0.03	-0.01
A3-N	2.25	NS	NS	NS	NS	NS
A6-N	NS	-0.07	NS	0.02	NS	2.81

A8-N	-0.03	-0.01	-0.24	-0.00*	6.15	-0.86
A10-N	-0.01	0.02	0.01	-0.02	0.10	-0.25
B4-N	NS	-0.05	-0.24	-0.00*	NS	-0.86
B6-N	0.01	0.02	0.13	-0.33	0.36	0.42
B7-N	NS	0.86	0.44	0.21	0.58	0.77
B8-N	0.12	-0.23	-0.05	-0.11	-0.09	0.04
B9-N	0.03	-0.29	0.53	0.10	1.40	1.00
B11-N	0.01	NS	0.01	-0.02	0.60	-0.08
B12-N	0.25	NS	-0.10	0.98	NS	NS
C3-N	0.28	-0.19	0.12	ND	0.14	-0.10
C5-N	-0.00*	0.00*	0.10	0.04	-0.56	-0.54
C6-N	-0.00*	0.00*	0.00*	-0.02	0.04	-0.02
C7-N	NG	NG	0.54	0.01	2.19	-0.79
C8-N	0.03	-0.01	0.07	-0.02	ND	-1.61
C10-N	0.33 ± 0.03	NS	1.50 ± 0.48	4.49 ± 3.20	NS	NS
D1-N	0.08	0.15	2.77	-0.48	NG	NG
D3-N	NS	-0.01	NS	0.04	NS	NS
D8-N	1.67	0.04	0.94	-0.30	0.83	-0.10
D10-N	0.01	-0.09	0.02	-0.16	0.03	-0.32
D11-N	NS	-1.27	2.22	NS	5.26	-0.34
E3-N	NS	NS	NS	0.16	0.09	0.10
E4-N	NG	NG	0.13	-0.02	0.13	-1.43
E6-N	-0.08	0.39	0.30	-0.07	0.12	-0.20
F2-N	0.36	-0.07	0.08	-0.55	4.14	0.13
F3-N	0.58	NS	0.04	0.01	0.34	-0.21
F6-N	0.32	0.40	0.20	-0.07	0.17	0.31
F7-N	NS	-0.06	0.01	NS	4.26	-0.20
F8-N	NS	0.37	-1.35	NS	NS	9.05
F9-N	NS	0.14	NS	NS	0.50	2.81
F10-N	0.15	-0.05	0.03	-0.76	0.18	-0.02
F12-N	0.05 ± 0.04	0.25 ± 0.05	0.98 ± 0.16	-0.39 ± 0.19	1.63 ± 1.27	-0.22 ± 0.65
G2-N	NS	-0.00*	0.76	-0.29	0.98	-0.41
G3-N	NS	NS	NS	NS	NS	NS
H1-N	0.41	-0.10	-0.35	-0.15	2.36	ND
H9-N	-0.00*	0.00*	0.07	0.02	0.09	0.13

Table S4. Morphological characters measured on 28 strains of *P. seriata*, compared with measurements from Hasle & Lundholm [55]. For each strain mean and standard deviation is presented.

Strain	Valve shape	Fibulae / 10µm	Striae / 10µm	Poroids / µm	Poroid rows	Apical axis / µm	Transapical axis / µm
P. seriata (Hasle & Lundholm, 2005)	Asymmetric lanceolate	14-20	14-20	6-8	2-more, usually 4	91-160	4.6-8.0
A9-C	Asym., lanceolate	14.6 ± 0.7	15.0 ± 0.9	6.9 ± 0.6	2+2	116.0 ± 20.8	4.9 ± 0.3
A1-N	Asym., lanceolate	15.6 ± 0.7	15.3 ± 1.0	6.4 ± 0.5	2+1-2	116.2 ± 3.6	5.5 ± 0.3
A6-N	Asym., lanceolate	14.2 ± 0.7	15.2 ± 0.8	6.2 ± 0.4	2+1-2	112.3 ± 14.8	5.7 ± 0.4
A8-N	Asym., lanceolate	14.6 ± 1.6	16.3 ± 0.5	6.4 ± 0.5	2+1-2	118.1 ± 13.5	5.2 ± 0.1
A10-N	Asym., lanceolate	14.1 ± 1.1	15.2 ± 1.0	6.3 ± 0.5	2+2	132.0 ± 17.6	5.6 ± 0.3
B6-N	Asym., lanceolate	18.0 ± 1.0	18.6 ± 1.1	7.7 ± 0.9	2+1-2	83.9 ± 8.1	4.3 ± 0.2
B9-N	Asym., lanceolate	14.7 ± 0.7	14.8 ± 1.0	6.6 ± 0.5	2+1-2	108.0 ± 4.6	5.8 ± 0.3
B11-N	Asym., lanceolate	16.1 ± 0.9	16.2 ± 0.8	6.8 ± 0.7	2+1-2	109.4 ± 2.2	5.0 ± 0.1
C5-N	Asym., lanceolate	14.2 ± 1.3	15.4 ± 2.4	7.3 ± 0.5	2+2	100.9 ± 18.6	5.2 ± 0.2
C6-N	Asym., lanceolate	15.0 ± 1.1	16.1 ± 1.3	6.7 ± 0.5	2+1-2	105.2 ± 5.8	6.1 ± 0.1
C7-N	Asym., lanceolate	12.8 ± 0.8	13.9 ± 0.6	6.1 ± 0.3	2+1-2	133.2 ± 24.2	5.7 ± 0.4

C8-N	Asym., lanceolate	14.6 ± 1.0	14.9 ± 0.8	6.9 ± 0.3	2+2	110.2 ± 13.5	5.4 ± 0.3
C10-N	Asym., lanceolate	15.1 ± 1.5	16.4 ± 1.1	6.3 ± 0.5	2+1-2	112.6 ± 3.6	5.2 ± 0.4
D1-N	Asym., lanceolate	15.5 ± 0.8	14.8 ± 0.9	7.0 ± 0.7	2+2	96.3 ± 14.1	5.1 ± 0.1
D3-N	Asym., lanceolate	16.8 ± 0.8	17.6 ± 1.0	6.9 ± 0.6	2+2	107.3 ± 3.3	5.4 ± 0.2
D10-N	Asym., lanceolate	13.9 ± 1.1	14.3 ± 0.7	6.4 ± 0.5	2+2	112.9 ± 10.5	5.8 ± 0.2
D11-N	Asym., lanceolate	15.1 ± 0.6	15.1 ± 1.3	6.4 ± 0.7	2+1-2	103.2 ± 8.1	5.5 ± 0.2
E3-N	Asym., lanceolate	15.8 ± 0.7	16.1 ± 0.6	7.0 ± 0.5	2+2	105.1 ± 5.6	5.0 ± 0.2
E4-N	Asym., lanceolate	15.4 ± 1.0	16.6 ± 0.9	6.9 ± 0.6	2+1-2	130.4 ± 10.3	5.6 ± 0.2
E6-N	Asym., lanceolate	16.0 ± 1.5	16.6 ± 1.1	6.7 ± 0.5	2+2	99.2 ± 6.1	5.1 ± 0.3
F6-N	Asym., lanceolate	13.3 ± 0.9	13.7 ± 0.9	6.3 ± 0.5	2+2	115.6 ± 6.8	5.5 ± 0.2
F7-N	Asym., lanceolate	13.6 ± 0.7	14.7 ± 0.7	6.3 ± 0.5	2+1-2	126.9 ± 11.0	6.4 ± 0.2
F10-N	Asym., lanceolate	15.6 ± 1.3	16.3 ± 1.1	6.4 ± 0.5	2+1-2	112.9 ± 2.6	5.4 ± 0.1
F12-N	Asym., lanceolate	14.0 ± 0.7	14.7 ± 2.1	6.4 ± 0.5	2+2	117.3 ± 0.4	5.7 ± 0.2
G2-N	Asym., lanceolate	13.7 ± 0.9	14.7 ± 1.0	6.6 ± 0.5	2+2	101.6 ± 3.9	5.6 ± 0.4

G3-N	Asym., lanceolate	14.7 ± 1.2	14.8 ± 1.4	6.4 ± 0.5	2+2	113.7 ± 16.5	5.6 ± 0.2
H1-N	Asym., lanceolate	13.9 ± 0.9	14.1 ± 0.9	6.9 ± 0.6	2+1-2	117.8 ± 2.6	5.6 ± 0.3
H9-N	Asym., lanceolate	15.1 ± 0.9	14.8 ± 1.0	6.6 ± 0.7	2+2	98.7 ± 5.3	5.2 ± 0.3
Mean		14.9 ± 1.1	15.4 ± 1.1	6,6 ± 0.4	2+1-2 / 2+2	111.3 ± 11.1	5.4 ± 0.4
